# Syncytin 1 dependent horizontal transfer of marker genes from retrovirally transduced cells

**DOI:** 10.1038/s41598-019-54178-y

**Published:** 2019-11-27

**Authors:** Berna Uygur, Kamran Melikov, Anush Arakelyan, Leonid B. Margolis, Leonid V. Chernomordik

**Affiliations:** 1 Section on Membrane Biology, Eunice Kennedy National Institute of Child Health and Human Development, National Institutes of Health, Bethesda, MD USA; 2 Section of Intercellular Interactions, Eunice Kennedy National Institute of Child Health and Human Development, National Institutes of Health, Bethesda, MD USA

**Keywords:** Genetic transduction, Membrane fusion

## Abstract

Retroviral transduction is routinely used to generate cell lines expressing exogenous non-viral genes. Here, we show that human cells transduced to stably express GFP transfer GFP gene to non-transduced cells. This horizontal gene transfer was mediated by a fraction of extracellular membrane vesicles that were released by the transduced cells. These vesicles carried endogenous retroviral envelope protein syncytin 1 and essentially acted as replication-competent retroviruses. The ability to transfer the GFP gene correlated with the levels of syncytin 1 expression in the transduced cells and depended on the fusogenic activity of this protein, substantiating the hypothesis that endogenous syncytin 1 mediates fusion stage in the delivery of extracellular vesicle cargo into target cells. Our findings suggest that testing for replication-competent retroviruses, a routine safety test for transduced cell products in clinical studies, should be also carried out for cell lines generated by retroviral vectors in *in vitro* studies.

## Introduction

Horizontal transfer of genetic materials, such as microRNAs, mRNA and DNA, between somatic mammalian cells occurs by a number of different mechanisms involving exogenous and endogenous retroviruses^1^, extracellular vesicles (EVs), including apoptotic bodies^2–4^, and even entire cells^5^, as well as tunneling nanotubes^6^. Horizontal transfer of the genetic material induces important changes in the properties of the recipient cells such as their viral resistance^3^.

Gene transfer mechanisms based on retroviral machinery are widely used both *in vitro* and *in vivo* to introduce genes of interest into mitotic cells. Retroviral vectors and cells containing retroviral vectors are considered for clinical applications^7^. Retroviral vectors approved for clinical applications and commercially approved retrovirus-based transduction systems are optimized to effectively deliver the gene and to keep the gene expressed in the progeny of the transduced cells. It is also critically important to minimize the risk of the production of replication-competent retrovirus (RCR) that may deliver the introduced gene or other genes from the transduced cell to non-transduced cells. To satisfy the latter requirement, the gene transfer plasmid lacks the genes required for γ-retroviral packaging and transduction. During production of retroviral vector these genes are provided by other plasmids or are stably expressed in the packaging cell line. Nevertheless, RCRs represent an important safety concern in the development of retroviral gene therapy^8^.

This study has developed from our serendipitous observation of double labelled cells in cultures of cells transduced with retroviral vector to express GFP co-plated together with cells transduced to express RFP. We found that emergence of double labelled cells reflects horizontal transfer of GFP gene between the cells and used this experimental system to explore the mechanism of this transfer. We report that this transfer depends on a cell type and is mediated by extracellular membrane vesicles (EMVs) that carry syncytin 1 (Syn1), endogenous fusion protein of retroviral origin expressed in placenta and at lower levels in many other tissues. Our findings suggest that testing for RCRs, a routine for transduced cell products in clinical studies, should be also carried out for cell lines generated by retroviral vectors in *in vitro* studies.

## Results

**Figure 1:**
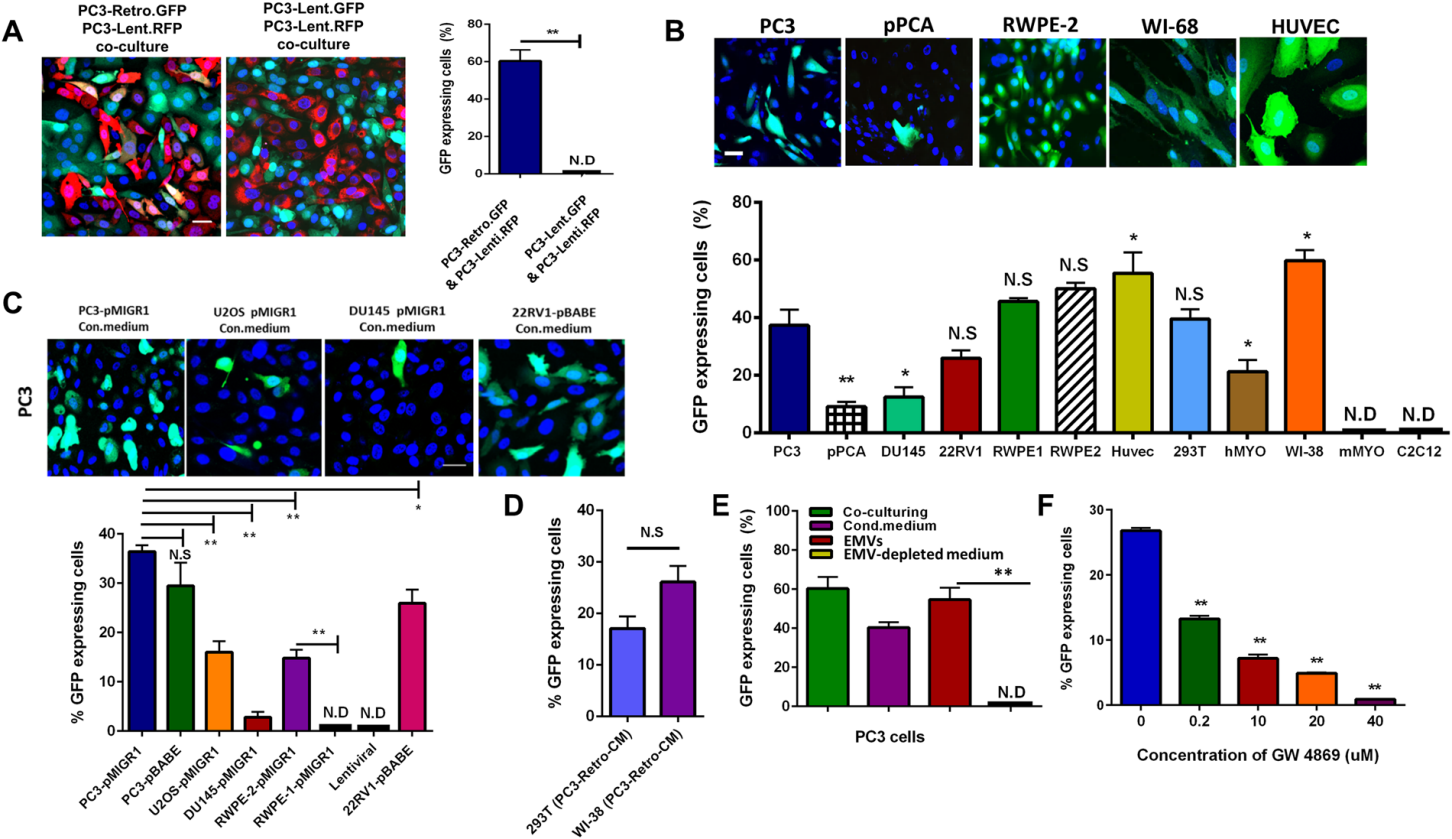
Transfer of GFP gene from retrovirally-transduced cells to non-transduced cells mediated by EMVs released into medium. (**A**) Representative images and quantification of GFP gene transfer from GFP-retro PC3 cells to RFP-lenti PC3 cells after 48 h co-culturing. **(B)** Representative images and quantification of GFP transfer to cells of different origin after culturing them in the conditioned medium from GFP-retro PC3 cells for 48 h. **(C)** Representative images and quantification of GFP transfer to PC3 cells after culturing them for 48 h in the conditioned media from different GFP-retro cells. **(D)** 293 T and WI38 cells were incubated in the conditioned medium from GFP-retro PC3 cells for 48 h. Then, the cells were washed with PBS and further cultured in fresh medium for 48 h. The conditioned media from these cells were used to culture non-transduced PC3 cells for additional 48 h. (**E**) Efficiency of GFP transfer into non-transduced PC3 cells after 48 h of: (1) co-culturing with GFP-retro PC3 cells; or incubation with (2) conditioned medium from GFP-retro PC3 cells, (3) EMVs isolated from this conditioned medium, or (4) EMV-depleted conditioned medium. (**F**) Dose dependence of GW4869 inhibition of GFP transfer by EMVs isolated from GFP-retro cells. **A**-**F**. All results are shown as means ± SEM (*n* ≥ 3). Levels of significance are shown as non-significant (NS), * and ** for p > 0.05, p < 0.05 and p < 0.005, respectively. The comparisons are shown relative to PC3 cells (B), EMVs from untreated PC3 cells (F) or indicated by lines. A,B,C Scale bar, 25 μm.

During our research related to prostate cancer cell fusion^9^, 48 hours after co-plating PC3 human prostate cancer cells transduced using lentiviral vector to express RFP (RFP-lenti) with PC3 cells transduced using pMIGR1-GFP retroviral construct to express GFP (GFP-retro) almost 60% of RFP expressing cells also expressed GFP (Fig. 1A). Independently, prior to our work, spreading of marker gene expression from retrovirally transduced cells to non-transduced cells has been described by Dr. Yuri Lazebnik in his report on a grant from the U.S. Army Medical Research and Materiel Command (https://apps.dtic.mil/dtic/tr/fulltext/u2/a501720.pdf). Using qPCR, we verified that this spreading of the GFP expression reflected delivery of GFP gene into RFP-lenti cells (Fig. S1). Similar transfer of the marker gene was also observed after co-incubation of RFP-retro with GFP-lenti PC3 cells (not shown). In contrast, cells co-expressing GFP and RFP were not observed if both GFP and RFP were expressed using lentiviral constructs (Fig. 1A). Only cells transduced with retroviral vector served as ‘donor’ cells, i.e., spread the expression of a marker gene to ‘acceptor’ cells.

GFP gene transfer was observed even when co-culturing of the cells was replaced with application of the conditioned medium from GFP-retro PC3 cells (Fig. 1B). The ability to serve as acceptor cells in the gene transfer was not limited to PC3 cells. The list of tested cells included different human cancer cells (PC3, DU145, 22RV1, pPCA); normal human embryonic lung cell line, WI-38; normal human prostate epithelial cells RWPE-1 and 2; normal embryonic kidney cells, 293T; human primary skeletal muscle cells (hMYO); human umbilical vein endothelial cells (HUVEC); as well as murine myoblasts (primary and immortalized cell line C2C12). All human cells that we tested developed GFP expression after 48 h incubation in the conditioned medium from GFP-retro PC3 cells, albeit with different efficiency (Fig. 1B). In contrast, murine myoblasts did not pick up GFP at all.

Not only the acceptor cells but donor cells as well were not limited to one cell line. We transduced bone osteosarcoma cells (U2OS), low metastatic prostate cancer cells (DU145), and RWPE-1 and 2 cells with pMIGR1 retroviral construct to stably express GFP. While conditioned media from U2OS and RWPE-2 effectively transferred GFP expression to PC3 cells, conditioned media from DU145 and RWPE-1 demonstrated inefficient transfer (Fig. 1C).

GFP gene transfer was not specific to using pMIGR1 retroviral construct. Conditioned media from PC3 cells and another prostate cancer cell line 22RV1 transduced with pBABE-GFP retroviral construct also transferred GFP gene to non-transduced PC3 cells (Fig. 1C).

We found that conditioned medium from the cells that acquired GFP expression by the gene transfer can then further transfer GFP expression to other non-transduced cells (Fig. 1D). As seen in Fig. 1B, non-transduced 293T cells and WI-38 treated with conditioned medium from GFP-retro PC3 cells demonstrated high levels of GFP expression. In turn, the conditioned media from these cells were used to treat non-transduced PC3 cells leading to the GFP expression in PC3 cells. This second round of spreading the GFP expression suggested the involvement of RCR.

Apparently, all mammalian cells release EVs. Since physical properties and sizes of many EVs strongly overlap with those of retroviruses^10–13^, for retrovirus-releasing cells, conventional EV preparations contain both EVs and retroviral particles and below will be referred to as extracellular membrane vesicles (EMV). To test whether the gene transfer depends on EMVs, we collected the conditioned medium in which GFP-retro cells were grown for 72 h and isolated the fraction of EMVs using ultracentrifugation. Western blot analysis of several markers of EVs, including TSG101, CD63, HSP70, CD54 and flotillin^14^, as well as particle count and size distribution analysis indicated that retroviral transduction of PC3 cells does not significantly change the numbers and sizes of EMVs released into the medium in comparison to those released by the non-transduced PC3 cells (Fig. S2A–C). EMVs isolated from the conditioned medium generated by GFP-transduced PC3 cells carried both GFP and GFP gene (Fig. S1B,C).

Non-transduced PC3 cells incubated with EMVs from GFP-retro cells developed expression of GFP, with the fraction of the GFP-expressing cells similar to the fractions observed after co-culturing the cells with GFP-retro cells or after application of the conditioned medium from these cells (Fig. 1E). No gene transfer was observed when non-transduced PC3 cells were incubated in the EMV-depleted conditioned medium from GFP-retro cells. EMVs isolated using another experimental protocol, in which vesicles were precipitated with polymer-based reagents, also mediated gene transfer (see below).

The dependence of the gene transfer on EMVs was further confirmed by finding that GW4869, an inhibitor of neutral sphingomyelinase, shown to inhibit budding and release of EV^15^ and viral egress^16^ inhibited the gene transfer (Fig. 1F).

**Figure 2:**
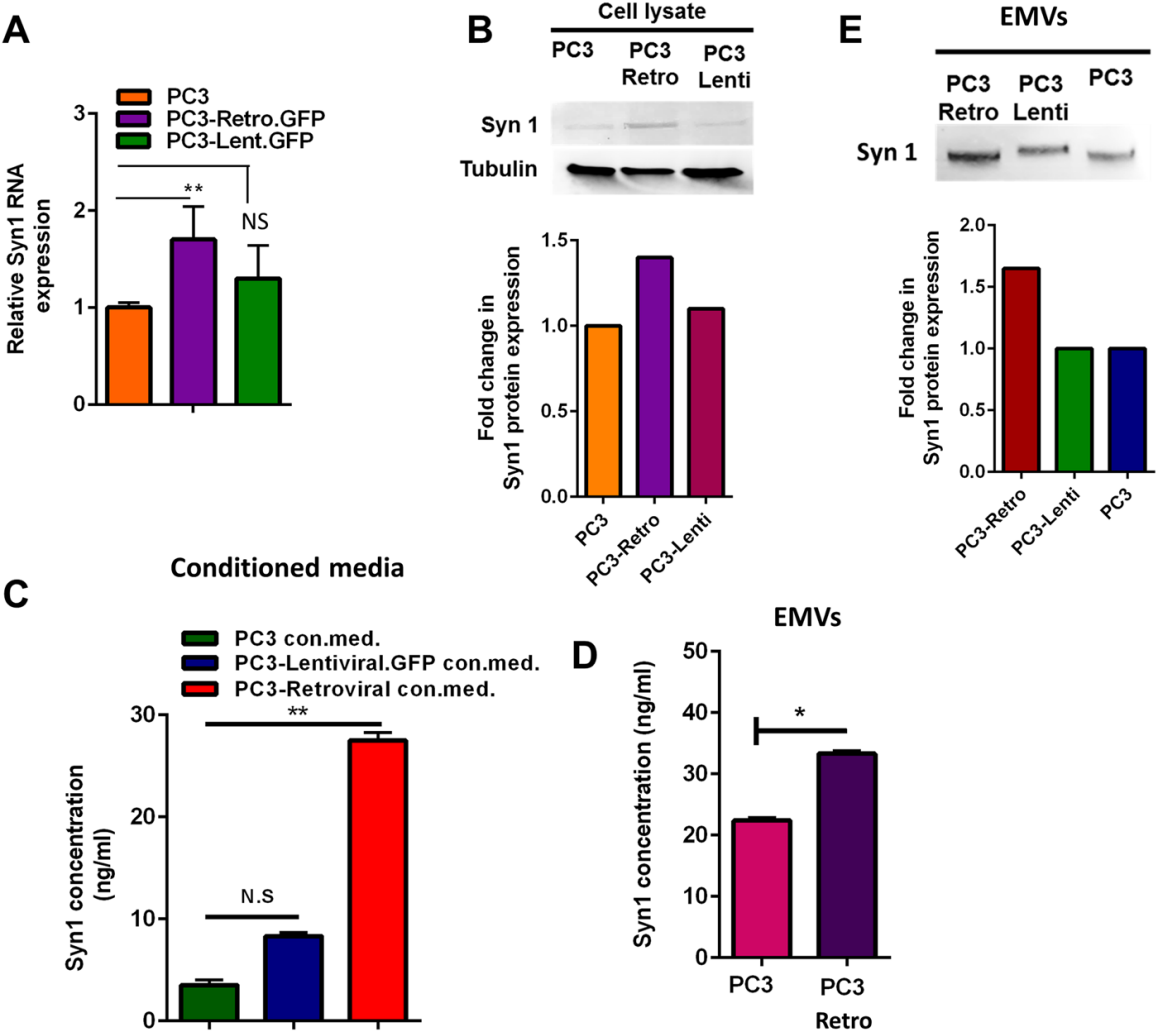
Syncytin 1 is expressed in GFP-retro PC3 cells and found in the conditioned medium and EMVs. (**A**,**B**) qPCR (**A**) and western blot (**B**) analysis of protein level and mRNA level of Syn1 in non-transduced, GFP-retro and GFP-lenti PC3 cells. Band intensity quantification relative to tubulin is shown under the gel. (**C**) Comparison of Syn1 protein level in the conditioned media from non-transduced, GFP-retro and GFP-lenti PC3 cells using ELISA assay. (**D**) ELISA analysis of Syn1 expression in EMVs from non-transduced and GFP-retro PC3 cells. (**E**) Western blot analysis of Syn1 expression in EMVs from non-transduced, GFP-retro and GFP-lenti PC3 cells. Band intensity quantification is shown under the gel. **A,C,D**. All results are shown as means ± SEM (*n* ≥ 3). Levels of significance are shown as non-significant (NS), * and ** for p > 0.05, p < 0.05 and p < 0.005, respectively. The comparisons are indicated by lines.

The results discussed above indicated that the gene transfer is mediated by EMVs released by retrovirally transduced cells. It has been proposed that EV-target cell fusion that delivers EV content into the cell can be mediated by endogenous retroviral envelope protein Syn1^17,18^. Interactions between Syn1 and its ubiquitous receptor ASCT2 trigger restructuring of Syn1 that brings about membrane fusion essential for formation of multinucleated syncytiotrophoblasts in placenta^19^. To examine whether EMVs involved in the gene transfer carry Syn1, we first compared Syn1 expression levels in lysates of non-transduced PC3 cells, GFP-retro and GFP-lenti PC3 cells (“PC3-retro” and “PC3-Lenti” in the figure) and found both RNA and protein expression to be slightly higher in GFP-retro PC3 cells than in other cells (Fig. 2A,B). ELISA analysis of the conditioned media from the non-transduced PC3 cells, GFP-lenti and GFP-retro PC3 cells showed the latter to have higher amounts of Syn1 than the former (Fig. 2C). Using ELISA and western blot analyses, we found that EMVs isolated from the conditioned medium from GFP-retro PC3 cells carried slightly more Syn1 than EMVs isolated from the conditioned medium from GFP-lenti PC3 and non-transduced PC3 cells (Fig. 2D,E). These findings confirmed the presence of Syn1 in EMVs released by GFP-retro PC3 cells.

**Figure 3:**
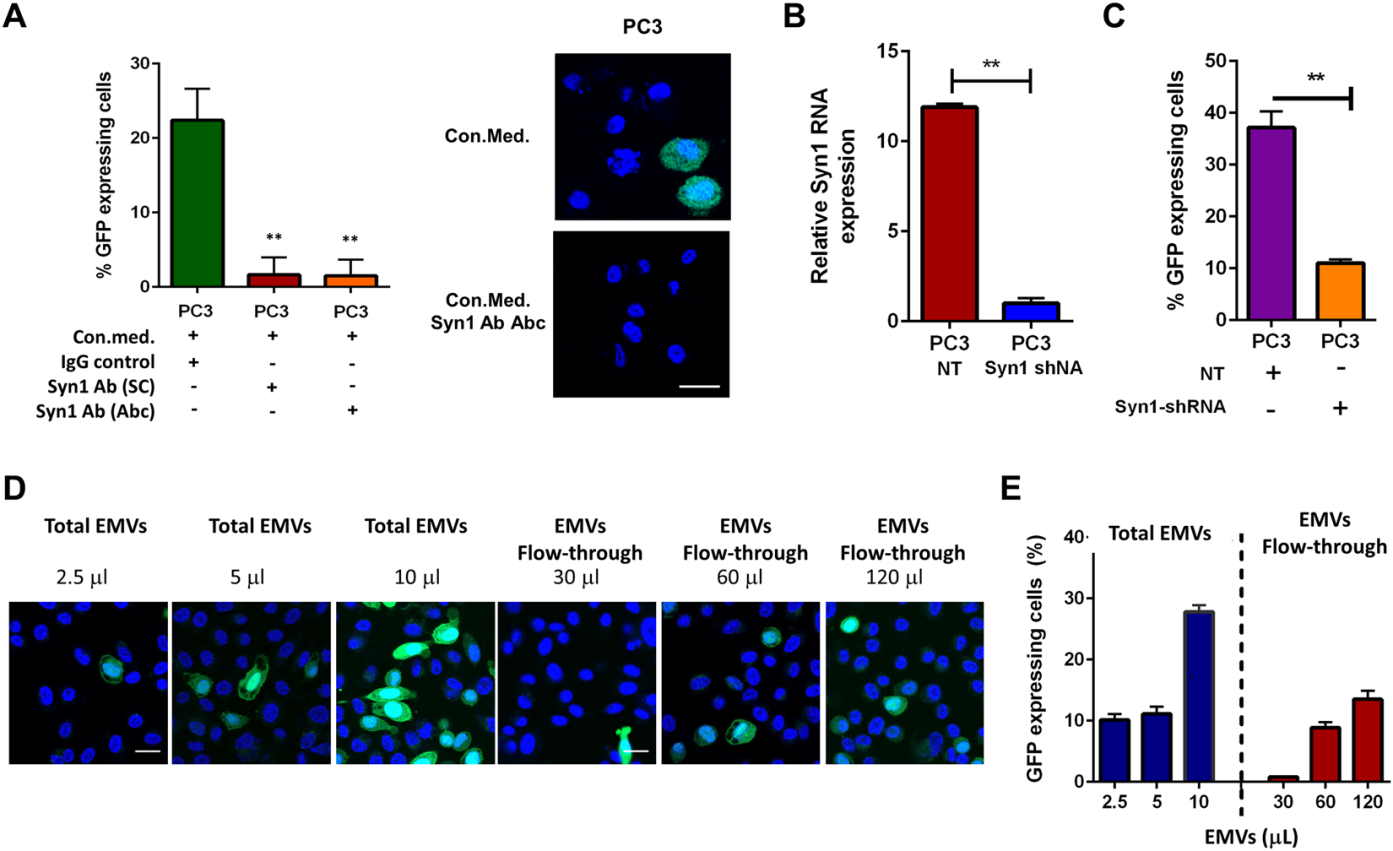
The gene transfer from GFP-retro PC3 cells depends on Syncytin 1. (**A**) Two different Syn1 antibodies inhibit the gene transfer by the conditioned medium from GFP-retro PC3 cells. **(B, C)** Expression of Syn1 targeting shRNA but not non-targeting (NT) shRNA in GFP-retro PC3 cells knocks down Syn1 expression, as confirmed by qPCR (**B**), and lowers the ability of the conditioned medium from these cells to mediate the GFP gene transfer into non-transduced cells (**C**). (**D**,**E**) Representative fluorescence images (**D**) and quantification (**E**) show less efficient GFP transfer for GFP-retro PC3 cell-derived EMVs depleted from Syn1 carrying vesicles. Scale bar, 25 μm. All results are shown as means ± SEM (*n* ≥ 2). Levels of significance relative to the data for the conditioned medium from GFP-retro cells expressing (**B**,**C**) or not expressing NT shRNA (**A**) are shown as ** for p < 0.005.

We then used several independent experimental approaches to examine the functional role of Syn1 in the gene transfer. Adding to the conditioned medium from the GFP-retro PC3 cells either of the two different antibodies to Syn1 inhibited the GFP expression transfer to the non-transduced PC3 cells (Fig. 3A). In another approach, we knocked down Syn1 expression in the GFP-retro PC3 cells with Syn1 shRNA using non-targeting (NT) shRNA as a control and confirmed efficiency of Syn1 knockdown with qPCR (Fig. 3B). We found the conditioned medium from GFP-retro PC3 cells with suppressed expression of Syn1 to be less effective in the GFP gene transfer (Fig. 3C). Silencing Syn1 expression in GFP-retro PC3 cells with Syn1 siRNA, confirmed by western blotting, also suppressed the GFP gene transfer to non-transduced PC3 cells (Fig. S3A) and non-transduced DU145 (Fig. S3B). This finding suggested that the transfer of the GFP gene is mediated by membrane vesicles carrying Syn1.

This conclusion has been further confirmed by experiments in which we first isolated EMVs generated by GFP-retro PC3 cells using polymer-based reagents and vesicle precipitation and then incubated these EMVs with magnetic nanoparticles coupled to anti-Syn1 antibodies. Syn1-carrying EMVs were captured on columns attached to magnet and the EMV fraction that was not retained (“flow through fraction”) was depleted of Syn1-carrying EMVs. Analysis of the concentrations of EMVs before separation and in flow-through fraction with nanoparticle tracking assay indicated that two thirds of EMVs generated either by GFP-retro PC3 cells or by non-transduced PC3 cells carry Syn1 (Fig. S2B,C). We found that EMV population depleted of Syn1 vesicles has considerably lower ability to transfer GFP gene (Fig. 3D,E).

**Figure 4:**
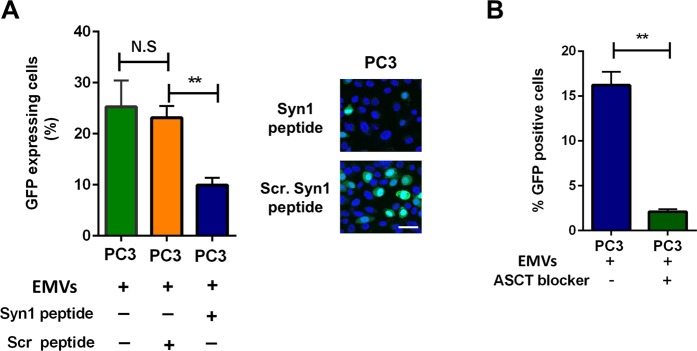
GFP gene transfer depends on fusion mediated by ASCT2 dependent fusogenic restructuring of Syncytin 1. Suppressing Syn1 restructuring with a peptide inhibitor (50 μg/ml) with its scrambled analog used as a negative control **(A)** and blocking Syn1 receptor ASCT2 with 35 μl/ml ASCT2-binding reagent **(B)** inhibit the gene transfer into non-transduced PC3 cells mediated by the conditioned medium from GFP-retro PC3 cells. All results are shown as means ± SEM (*n* ≥ 3). Levels of significance relative to the data for EMVs from GFP-retro PC3 cells in the presence of scrambled Syn1peptide (**A**) or EMVs from GFP-retro PC3 cells (**B**) are shown as non-significant (NS) and ** for p > 0.05 and p < 0.005, respectively. The comparisons are indicated by lines.

To verify that the gene transfer depends on the fusogenic activity of Syn1 rather than on its fusion-unrelated functions^20^, we used a Syn1-derived synthetic peptide known to block fusogenic restructuring of Syn1^21^. We found this peptide but not the control scrambled peptide to suppress the GFP transfer by EMVs collected from GFP-retro PC3 cells and applied in the presence of the peptides to non-transduced PC3 cells (Fig. 4A). Furthermore, if the gene transfer depends on the fusogenic activity of Syn1, one would expect blocking ASCT2 receptor of Syn1 with an ASCT2-binding reagent ASCT2.RBD to also suppress the gene transfer. Indeed, application of this reagent inhibited GFP gene transfer by the EMVs from GFP-retro PC3 cells to the non-transduced PC3 and HUVEC cells (Figs. 4B and S4). These finding indicated that the GFP gene transfer depends on Syn1-mediated fusion.

**Figure 5:**
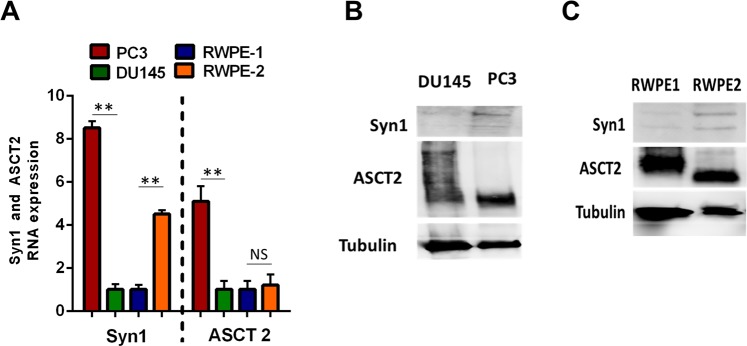
A higher ability to serve as donor cells correlates with higher levels of Syncytin 1 expression. (**A**) Syn1 and ASCT2 expression at the RNA level in PC3, DU145, RWPE1 and RWPE2 cells measured with qPCR. All results are shown as means ± SEM (*n* ≥ 3). Levels of significance in comparison between different cancer cells and different normal cells are shown as NS for p > 0.05 and ** for p < 0.005. (**B, C**) Syn1 and ASCT2 expression in DU145 and PC3 (**B**) and in RWPE1 and RWPE2 cells (**C**) at the protein level evaluated with western blotting using tubulin as a loading control.

To explore the factors that determine the relative abilities of different cells to serve as donors and acceptors, we focused on two prostate cancer cell lines: high metastatic PC3 and low metastatic DU145; and two normal prostate epithelial cells: RWPE-1 and 2 (Fig. 5). First we noted that out of two cancer cell lines, PC3 cells that we found to be better donors (Fig. 1C) express higher amounts of Syn1 (Fig. 5A,B). Similarly, among two non-cancerous cell lines, better donors RWPE-2 expressed more Syn1 than RWPE-1 (Fig. 5A,C). We conclude that the relative ability of the cells to spread the GFP gene correlates with Syn1 expression (PC3 more efficient donors than RWPE-2 more efficient than DU145 and RWPE-1), and normal cells expressing high levels of Syn1 (e.g., RWPE-2) can be better donors than cancer cells that express relatively low amounts of Syn1 (e.g., DU145). The lack of correlation between levels of ASCT2 expression and the ability of the cells to serve as either acceptors or donors suggest that even cells expressing lower amounts of ASCT2 carry enough receptor to support Syn1-mediated fusion of EMV.

**Figure 6:**
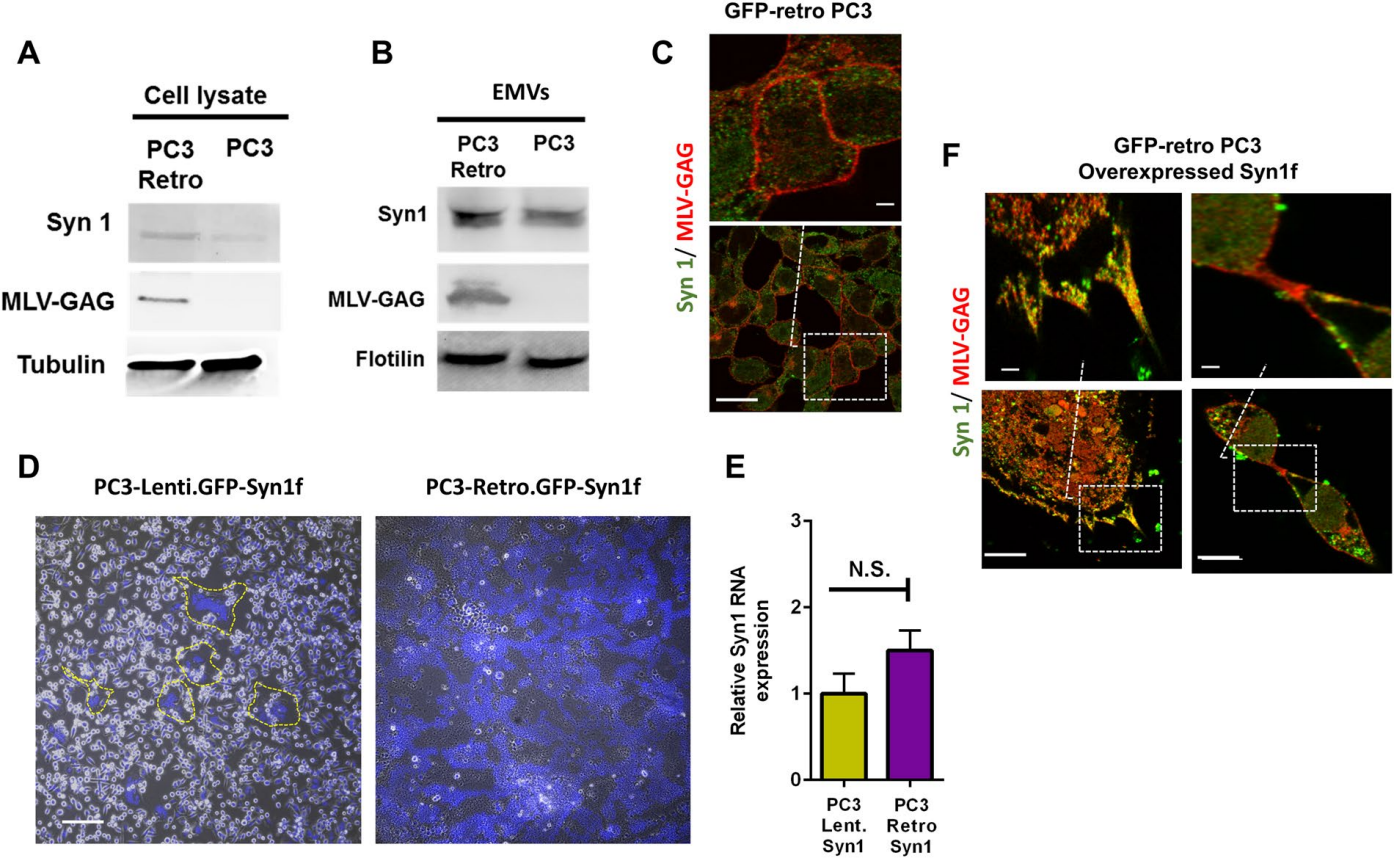
MLV-GAG is expressed in the retrovirally transduced cells supporting Syn1-dependent gene transfer. (**A**,**B**) Western blot analysis of MLV-GAG and Syn1 expression in the lysates of GFP-retro PC3 cells and non-transduced PC3 cells (**A**) and in the EMVs generated by these cells (**B**). **(C**) a representative image of GFP-retro PC3 cells labeled with antibodies to Syn1 (Alexa Fluor 405 labeled secondary antibodies shown in green) and MLV-GAG (Alexa Fluor 633 secondary antibodies shown in red) selected out of 10 fields in 3 experiments. (**D**) Representative fluorescence microscopy images of GFP-retro PC3 and GFP-lenti-PC3 cells transfected to express hyperfusogenic Syn1f. In the left image, yellow outlines depict the boundaries of syncytia. In the right image, a single syncytium covers the entire field of view. Scale bar 25 μm (**E**) GFP-retro PC3 and GFP-lenti PC3 cells, both transfected to express Syn1f have similar total (including both endogenous Syn1 and Syn1f) levels of Syn1 mRNA. All results are shown as means ± SEM (n = 3) and the level of significance is shown as NS for p > 0.05. (**F**) Representative fluorescence microscopy images of Syn1f-expressing GFP-retro PC3 cells labeled with antibodies to Syn1 (Alexa Fluor 405 labeled secondary antibodies shown in green) and MLV-GAG (Alexa Fluor 633 secondary antibodies shown in red). Images at the top show enlargements of outlined regions. Scale bar 50 μm.

Although efficiency of gene transfer from retrovirally transduced cells correlated with the level of Syn1 expression, overexpression of Syn1 in cells transduced with lentiviral GFP/RFP constructs did not induce gene transfer (Fig. S5). These finding suggest that the transfer depends on some retroviral proteins. Indeed, surprisingly, since GFP gene was delivered into donor cells by a replication-defective retroviral vector, we found GFP-retro PC3 cells and EMVs released by GFP-retro PC3 cells to contain MLV GAG (Fig. 6A,B). Neither non-transduced PC3 cells nor EMVs generated by non-transduced cells contained MLV GAG, suggesting that MLV GAG in EVs is encoded in retroviral construct and, by extension, that the transfer involves RCR. Immunofluorescence microscopy detected MLV GAG expression along plasma membrane of the cells (Fig. 6C). We hypothesized that interactions with MLV GAG promote Syn1 mediated fusion, possibly by raising local concentrations of Syn1. This hypothesis has been substantiated by our experiments, in which we transfected GFP-lenti PC3 cells to express exogenous Syn1 with a fusion-enhancing deletion in the cytoplasmic domain (Syn1f)^22^ and, as reported earlier^9^, observed syncytium formation (Fig. 6D, image on the left). Notably, GFP-retro PC3 cells transfected to express Syn1f (Fig. 6D, image on the right) formed syncytia much more efficiently than GFP-lenti PC3 cells, despite comparable Syn1f expression levels (Fig. 6E). Immunofluorescence microscopy with antibodies to MLV GAG and Syn1 indicated that MLV GAG and Syn can be found in the same regions of the membranes including cell-cell contacts (Fig. 6F). The striking difference in the extents of Syn1 mediated cell-cell fusion can reflect fusion promotion by Syn1 interactions with MLV GAG expressed in GFP-retro PC3 cells.

## Discussion

Diverse cell biological studies rely on retroviral transduction for *in vitro* generation of stable cell lines expressing a gene of interest or RNA molecules. It is well known that introducing the helper genes gag-pol and env on one plasmid rather than as separate transcriptional units increases the risks of recombination events generating RCRs^23^. However, to achieve very high levels of gene expression in a large fraction of the transduced cells, many *in vitro* studies utilize a single packaging vector encoding gag, pol and env such as pCL vector^24–26^. In this study we show that an efficient retroviral transduction of GFP into human cells with widely used transfer (MIGR1 and pBABE) and packaging pCL-Ampho vectors is accompanied by generation of RCRs. Expression of MLV GAG in the transduced cells and its presence in the EMVs released by these cells suggest recombination events between the vector and packaging constructs.

In our work, RCRs are detected as EMVs that carry the marker protein gene (GFP) and endogenous retroviral fusogen Syn1 that mediates EMV/cell fusion and, thus, delivers the marker gene into non-transduced human cells. Overexpression of Syn1 in the cells transduced with an envelope-defective retroviral sequences has been reported to result in assembly of pseudotyped viruses with the ability to fuse with cells and to spread viral gene sequences to new target cells^27^. Our findings indicate that even endogenous levels of expression of Syn1 in cells can be sufficient for this protein to be acquired by EMVs and to mediate Syn1-mediated fusion of these vesicles. Moreover, we found retroviral transduction of the cells to raise fusogenic potential of Syn1.

Fusogenic restructuring of Syn1 that we found to be required for the gene delivery by EMVs is triggered by interactions between Syn1 and its receptor, neutral amino acid transporter type 2 (ASCT2) expressed in human but not in murine cells, which do not support Syn1-mediated fusion^28^ and do not serve as acceptor cells (Fig. 1B). Importantly, the levels of the expression of both Syn1 and ASCT2 widely vary between different human cells and under different conditions. For instance, expression of Syn1 and ASCT2 is boosted by cytokines interleukins 4 and 13 and by tumor necrosis factor alpha^9,29^. Multiple types of cancer, including prostate adenocarcinoma and endometrial carcinoma, have elevated levels of Syn1 expression with further upregulation of Syn1 associated with cancer progression^9,29^. Syn1 expression is also boosted by some viral infections^30^ and linked to neurological diseases^31^. These data suggest that some retrovirally transduced cells can generate EMVs with Syn1-dependent infectivity only under specific conditions that boost Syn1 expression in these cells or ASCT2 expression in the surrounding cells. Indeed, we found the ability of the transduced cells to serve as donor cells to correlate with levels of expression of Syn1. Furthermore, Syn1 interactions with MLV GAG can increase surface density of Syn1 on the EMVs relative to the density on the surface of non-transduced cells. Importantly, the relative efficiency of infection for RCRs carrying Syn1 for different cell types depends on ASCT expression, while the relative efficiency of infection for the virus carrying the envelope protein of 4070A murine leukemia virus encoded in the pCL-Ampho vector depends on PIT-2 expression^25^. Analogously, the tropism of RCRs can be redirected for RCRs that carry and utilize envelope proteins of other endogenous retroviruses such as HERV-K and HERV-FRD.

Retrovirally transduced cells most likely generate both RCR particles and constitutively released EVs. RCRs that mediate the gene transfer from retrovirally transduced cells to non-transduced cells are isolated with EV isolation protocols. The release of these PCRs is suppressed by GW4869, an inhibitor of EV release. Like most EVs released by PC3 cells, RCRs carry endogenous retroviral protein Syn1. While RCR fusion is mediated by Syn1, as evidenced by inhibition of the gene transfer by a peptide inhibiting fusogenic restructuring of Syn1, a possible role of Syn1 in the delivery of EV cargo into cells has been suggested but remains to be proven^17,18,32^. All these similarities between RCR particles and EVs emphasize the overlap between the properties of retroviral particles and EVs and an ambiguity of the distinctions between RCRs and “constitutive” EVs that are released in a retroviral-transduction-independent way^11^. While throughout this paper we characterize the efficiency of the gene transfer by measuring a fraction of the cells that has received and expressed the marker gene GFP, in future work it will be interesting to characterize the fusogenic activity of Syn1 at EMVs by measuring the fraction of EMVs carrying both Syn1 and GFP gene that deliver their content into the acceptor cells. We expect future work to also clarify whether generation and function of the constitutive EVs depends on any endogenous retroviral genes and proteins.

In the context of clinical applications, where generation of RCRs is expected to increase the risk of treatment-related malignancy, emergence of RCRs is a very well recognized concern^8^. Both vector-producing cells and the transduced cells developed for gene therapy studies are routinely tested for the presence of RCR^8,33^. Our findings indicate that stable cell lines generated by retroviral vectors in *in vitro* studies, where the transduction protocols are optimized to achieve the highest efficiency of the transduction by utilizing single packaging vector encoding gag, pol and env, also must be tested for RCRs. Repetitive integration events that can accompany multiple infections by RCR can lead to important changes in protein expression profile and cell properties. Undetected and unexpected RCR production in laboratory cell lines presents an additional safety concern, especially, given the fact that in human cell line HERV env proteins can change the tropism of the produced viral particles.

## Methods

### Cell lines

PC3, DU145, 22RV1, U2OS, WI-38, RPEW1, RPEW2, HUVEC, 293T, CHO and C2C12 cells were obtained from American Type Culture Collection (ATCC, Manassas, VA), primary human myoblasts (hMYO), were obtained from Lonza and were maintained in the culture medium according to the manufacturer’s instructions. Primary human prostate cancer cells (pPCa) developed from a human prostate adenocarcinoma sample (Gleason 8 (4 + 4), T2N0M0) were obtained from Celther, Polska (Poland) and cultured according to the manufacturer’s instructions. Primary murine myoblasts were isolated and grown as in^34^.

### Reagents and antibodies

We used synthetic peptide inhibitor of Syn-1-mediated fusion (Syn-1 peptide, Ac-SGIVTEKVKEIRDRIQRRAEELRNTGPWGL-NH2), described and characterized in ^21^c, and as a negative control (Syn-1 scr peptide) a peptide with the same amino acid composition but a scrambled sequence (Ac-GKWGLSRIRTELRNTEPVKEQVRAEIGDRI-NH2). Both peptides were custom synthesized by GenScript. ASCT2 was blocked with ASCT2 receptor binding domain (ASCT2.RBC.MFc, Metafora). To block Syn1 dependent fusion, we also used Syncytin-1 (H-280) antibody from Santa Cruz (Cat# SC-50369), Syncytin 1 antibody from Abcam (Cat.# 71115), and, in control experiments, non-specific IgG (Cell signaling, Cat.# 2729). A neutral sphingomyelinase inhibitor GW4869 was purchased from Sigma Aldrich (Cat# D1692) and was used to block EMV budding.

### Plasmids

pMIGR1(Cat. # 27490) and pBABE (Cat. # 14430) were obtained from Addgene. pCL-Ampho (Cat#10046P) packaging plasmid with env gene from 4070A murine leukemia virus (MuLV) was obtained from Imgenex. Plasmid pHCMV HERV-Hyper-W expressing Syn1 mutant (Syn1f) with C-terminal truncation that increases the fusogenicity of the protein^22^ was a kind gift from Drs. Valery Krizhanovsky, The Weizmann Institute of Science, and Shmuel Rozenblatt, Tel Aviv University.

### Viral transduction

The 293T cells were plated at 3 × 10^5^ cells per well in a six-well plate, cultured overnight, and then transfected with a mixture of DNA containing 2.5 μg of pMIGR1 or pBABE and 1.5 μg of pCL-Ampho packaging plasmid (Imgenex) using MirusTrans-IT 2020 (Mirus) transfection reagent, according to the manufacturer’s instructions. The supernatant of the transfected cells was collected 24 h later and filtered through a 0.45- μm pore-size filter. For viral infection, PC3, DU-145, U20S, RWPE-1, RWPE-2 cells were seeded at 50% confluence in 6-well plates. The next day, the virus-containing supernatants from 293T cultures mixed with polybrene (Sigma, St. Louis, MO) at a final concentration of 4 mg/ml were added to the cells in each well. The plate was centrifuged at 1000xg for 1 hr at 35 °C. After discarding the medium, application of the virus-containing supernatant and centrifugation were repeated two more times. Then the cells were returned to the CO_2_ incubator. 72 h hour later the cells were examined for the GFP expression under the microscope.

### The gene transfer assay

To prevent a possible contamination with pseudoviruses used to generate the cells stably expressing retroviral or lentiviral GFP/RFP constructs, the cells were maintained using regular cell culturing procedures for at least two weeks after transduction. Then, the transduced cells were cultured in their original medium and conditioned medium was collected at the end of the 72 h. The target cells were seeded the night before the treatment at 70% confluency and then were cultured for 48 h in the mixture of 50 percent fresh medium and 50 percent conditioned medium from the transduced cells. To measure GFP gene transfer, cells were imaged using confocal microscopy.

The gene transfer to the non-transduced cells treated with conditioned medium or EMVs from the GFP-retro cells was detected as appearance of GFP expressing cells and quantified by dividing the number of nuclei in GFP expressing cells by the total number of nuclei within the field of view. In the co-culturing experiments, GFP-retro cells were co-cultured with RFP-lenti cells in 1:1 ratio for 48 h. Then the cells were imaged using confocal microscopy. The gene transfer was quantified as the ratio of the number of nuclei in double-labeled) cells to the total number of RFP expressing cells within the field of view.

### RNA isolation, reverse transcription, and qPCR analysis

RNA was extracted with the RNeasy Plus mini kit (Qiagen). cDNA was synthesized by random priming from 1 µg of total RNA with the Q-Script cDNA super mix kit (Quanto biology), according to the manufacturer’s protocols. Primers for qPCR analysis were synthesized by the Eurofins Scientific. We performed qPCR using PerfeCTa SYBR Green FastMix (Bio-Rad), according to the manufacturer’s protocol. We analyzed data, determining quantities of gene-specific mRNA expression, using the comparative CT method as described previously^35^. CT refers to the “threshold cycle” and is determined for each experiment with MyiQ software. Amplification of GAPDH was performed for each reverse-transcribed sample as an endogenous quantification standard. The primers are as follows: GFP, 5′-TCAAGAGTGCCATGCCTGAA-3′ and 5′-TGGTCTGCTAGTTGAACGCT-3′, Syn1 (both endogenous Syn1 and Syn1f), 5′-ATGGAGCCCAAGATGCA-3′ and 5′-AGATCGTGGGCTAGCAG-3′, ASCT2, 5′-CACCATGGTTCTGGTCTCCT-3′ and 5′-GCGGGTGAAGAGGAAGTAG-3′ and GAPDH, 5′-ATTGACCTCAACTACATGGTTTACATG-3′ and 5′-TTGGAGGGATCTCGCTCCTGGAAG-3′.

### Western-blot analysis

Cells were lysed in cell lysis buffer (0.5% Triton X-100, 20 mM Tris, 100 mM NaCl, 1 mM EDTA, 1 mM beta glycerophosphate (Boston BioProducts), and 1 mM sodium orthovanadate (Boston BioProducts), supplemented with protease inhibiter cocktail (Sigma), as suggested by manufacturer. The lysate was transferred into an Eppendorf tube, sonicated for 10 s, and centrifuged for 10 min at 15,000 rpm and 4 °C. The soluble fraction of the lysate was added to the denaturing sample buffer (Boston BioProducts), boiled, loaded onto a 4–12% Mini Protean TGX gel (Bio-Rad), and separated with SDS-PAGE. We transferred the separated proteins to a PVDF membrane (Bio-Rad) using the Bio-Rad Trans-blot turbo transfer system. After the transfer, the membrane was blocked with 5% nonfat dry milk dissolved in PBS containing 0.05% Tween 20 (Bio-Rad). The membrane was incubated at 4 °C overnight with the primary antibodies. After incubation, the membrane was washed three times with PBS containing 0.05% Tween 20 and then incubated with alkaline phosphatase–conjugated secondary antibody (Thermo Fisher Scientific) for 1 h. The membrane was washed three times with PBS containing 0.05% Tween 20, and the protein bands were visualized with enhanced chemifluorescence (ECF reagent; GE Healthcare). We used following antibodies: Syn 1 (Abcam, Cat. # Ab179693), GFP (Cell Signaling, Cat# 29565), TSG101(Novus, Cat# NB200-112), CD63 (Cell guidance system, Cat#CGS82X), HSP70 (Cell Signaling, Cat# 4876), CD54 (Cell Signaling, Cat# 4915), Flotillin (Cell Signaling, Cat# D2V7J), MLV-GAG (Abcam, Cat# Ab100970). The cropped gels shown in the figures are based on the full-length gels presented in Figs. S6–13.

### ELISA assay for Syncytin-1

Human Syncytin-1 (ERVWE1) ELISA kit from MyBioSource (Cat.# MBS9318236) was used to measure Syn1 protein level in the conditioned cell culture medium. Manufacturer’s protocol was followed as recommended. In brief, 50 µl Standard or samples were added to the corresponding wells and then 100 µl HRP-Conjugate Reagent was added to every well except the blank wells. Plate was incubated for 60 minutes at 37 °C. All wells were washed 4 times. Then, 50 µl Chromogen Solution A and 50 µl Chromogen Solution B were added to every well. Plate was mixed gently and incubated for 15 minutes at 37 °C. Lastly, 50 µl Stop Solution was added to every well and optical density (O.D.) was measured at 450 nm using plate reader within 15 minutes after adding stop solution.

### EMV extraction

We isolated EMVs using Exoquick-TC exosome isolation reagent (Cat# EXOTC50A-1) following manufacturer recommendations. In brief, conditioned cell culture medium was centrifuged at 3000 g for 15 minutes to remove cells and cell debris. Supernatant was mixed with the appropriate volume of Exoquick-TC and mixed well by inverting or flicking the tube. Mixture was refrigerated overnight at +4 °C. Exoquick-TC/medium mixture was centrifuged at 1500 g for 30 minutes. After centrifugation, the EMVs appeared as a beige or white pellet at the bottom of the vessel. Supernatant was removed and residual Exoquick-TC solution was spinned down by centrifugation at 1500 g for 5 minutes. EMV pellets were suspended in 100–500 µl sterile 1X PBS.

We have also extracted EMVs using differential ultracentrifugation protocol described previously^36^. In brief, collected supernatant was centrifuged at 300 g for 10 minutes to exclude cells, then the supernatant was taken and centrifuged at 2000g for 10 min to exclude dead cells. Next, the supernatant was centrifuged at 10,000 g for 30 min. Lastly, the supernatant was centrifuged at 100000 for 70 min to collect EMVs. then supernatant was removed, and EMVs were washed with 1X PBS and centrifuged at 100,000 g for 70 min. EMV pellets were suspended in sterile 1X PBS.

### Immunodepletion of Syn1-Carrying EMVs

15 nm iron oxide magnetic nanoparticles (MNPs) (Ocean NanoTech, Springdale, AR) were coupled to 0.5 mg anti-syncytin-1 ERVWE1 monoclonal antibody (M06), clone 4F10 (Abnova, catalogue # H00030816-M06) according to the manufacturer’s recommendations. After coupling anti-syncytin-1-MNPs were resuspended in 1 ml storage buffer and stored at 4 °C until use. To deplete Syn1 carrying EMVs from total EMV population isolated from the conditioned media from GFP-retro PC3 cells and non-transduced PC3 cells anti-Syn1-MNPs (~3.9 × 10^12^ particles in 60 µl) were incubated with EMV preparation (~7.15 × 10^10^ EMVs in 100 µl) at 4 °C for 1 hour. EMVs captured by anti-Syn1-MNPs were separated on magnetic columns, while the non-captured fractions (“flow-through”) were collected and used for western analysis and application to non-transduced PC3 cells treatment.

### Nanoparticle tracking analysis (NTA)

NanoSight (Salisbury, United Kingdom) NS 300 with NTA 3.0 software was used for EMV enumeration and size measurements. The measurements were performed with constant sample flow using a syringe pump for 3 × 60 sec at camera level 13.

### Immunofluoresence for Syn1 and MLV-GAG

To detect Syn-1 and MLV-GAG expression, we used murine anti-Syn1 antibody (Abnova, cat.#. H000308; 1:20 dilution) and rabbit MLV-GAG antibody (Abcam, cat.# 100970), respectively. Cells were washed with PBS and fixed with warm (37 °C) 4% formaldehyde in PBS (Sigma, F1268) for 20 min. After three washes with PBS without calcium and magnesium, the cells were incubated in 10% FBS in PBS without calcium and magnesium to block non-specific binding. All primary antibodies were applied to the cells, still in 10% FBS in PBS without calcium and magnesium and incubated overnight at 4 °C. After five washes with PBS, the cells were incubated for 1 h at room temperature with PBS, 10% FBS. Alexa Fluor 405-tagged goat anti-mouse antibodies (Thermo Fisher Scientific, Cat.# 31553) and Alexa Fluor 633-tagged goat anti-rabbit antibodies (Thermo Fisher Scientific, Cat#27040) were applied in PBS, 10% FBS in 1:400 dilution for 1 h at room temperature. Cells were washed five times and imaging was performed with confocal microscopy.

### Statistical analysis

We analyzed the data using the Student’s *t*-test (one-tailed). A *p*-value < 0.05 was used to define statistically significant differences.

## Supplementary information

Supplementary Materials

## Data Availability

The data used to support the findings of this study are available from the corresponding author upon request.
