# Supplementary material for: Syncytin 1 dependent horizontal transfer of marker genes from retrovirally transduced cells
Source: Sci Rep. 2019 Nov 27;9:17637. doi: 10.1038/s41598-019-54178-y (PMC6881383; doi:10.1038/s41598-019-54178-y)
Supplement: Supplementary file 1 — Supplementary Materials [file 41598_2019_54178_MOESM1_ESM.pdf]

Syncytin 1 dependent horizontal transfer of marker genes from retrovirally transduced cells

Berna Uygur[1], Kamran Melikov[1], Anush Arakelyan[2], Leonid B. Margolis[2], Leonid V. Chernomordik[1,*]

[1] Section on Membrane Biology and [2] Section of Intercellular Interactions, Eunice-Kennedy National Institute of Child Health and Human Development, National Institutes of Health, Bethesda, MD, USA.

[*] Correspondence and requests for materials should be addressed to LVC (email: chernoml@mail.nih.gov)

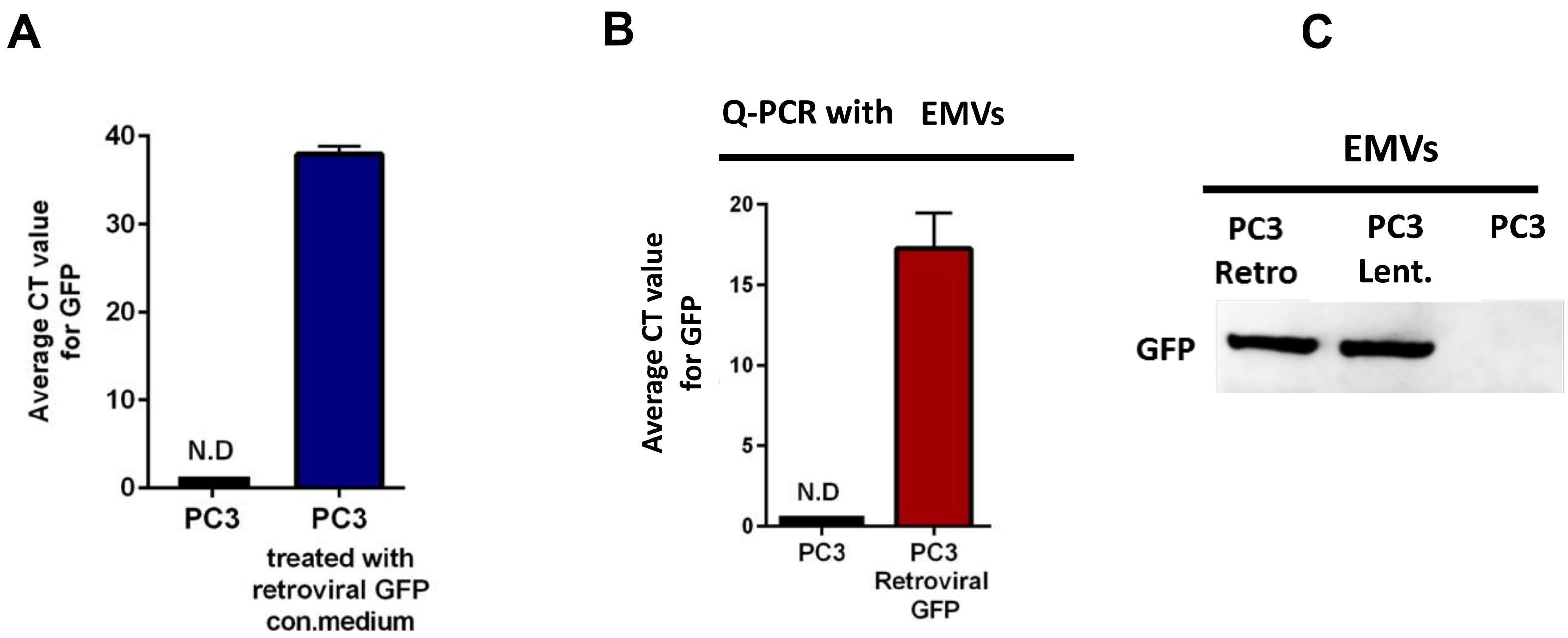

Figure S1. **GFP expression in EMVs and in non-transduced acceptor PC3 cells.** (**A**) GFP gene is detected in acceptor non-transduced PC3 cells incubated with the conditioned medium from GFP-retro PC3 cells. (**B**) GFP gene is present in EMVs generated by GFP-retro PC3 cells but not non-transduced PC3 cells. **(C)** Western blot analysis shows GFP presence in EMVs generated by GFP-retro and GFP-lenti PC3 cells but not by non-transduced PC3 cells. All results are shown as means ± SEM ($n \geq 3$).

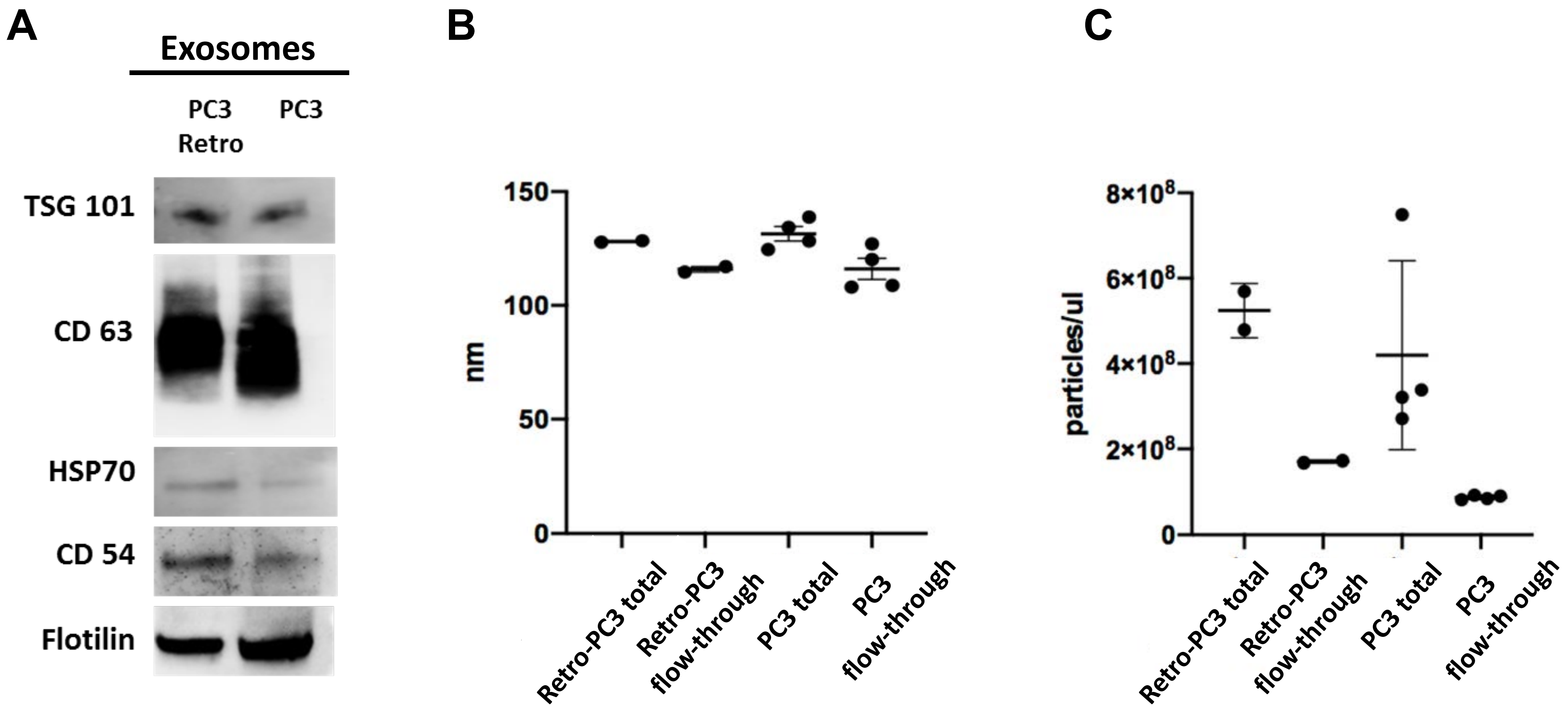

Figure S2. **Characterization of EMVs extracted from the conditioned medium generated by GFP-retro PC3 and non-transduced PC3 cells.** (**A**) Western blot analysis of EMVs for exosome markers TSG101, CD63, HSP70, CD54, and Flotillin. **(B, C)** Sizes and numbers of EMVs were measured using Nanosight before and after depletion of Syn1 expressing EMVs. Results of individual experiments are shown as dots. Means +/- SEM are shown as horizontal lines with whiskers. (**D, E**) Western blot analysis of Syn1 and MLV-GAG in EMVs before and after depletion of Syn1 expressing EMVs.

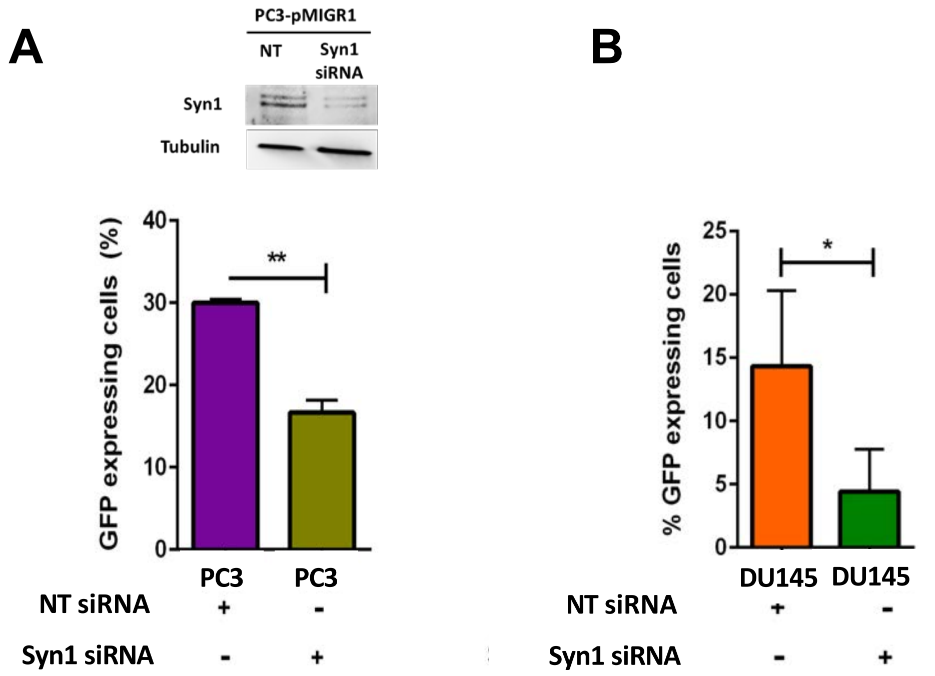

Figure S3. Silencing Syn1 in GFP-retro PC3 cells inhibits the gene transfer into non-transduced PC3 cells (A) and DU145 cells (B). (**A**) Protein level of Syn 1 in GFP-retro PC3 cells after silencing Syn1 expression with Syn1 siRNA. (**A, B**) Expression of siRNA targeting Syn1 but not of non-targeting (NT) siRNA inhibited gene transfer to both PC3 and DU145 cells. Insert in (A) shows western blot analysis of Syn1 levels in GFP-retro PC3 expressing Syn1 siRNA and NT siRNA with tubulin as a loading control. All results are shown as means ± SEM ($n \geq 3$). Levels of significance relative to the data for the conditioned media from GFP-retro PC3 cells are shown as * $p<0.05$ and ** for $p<0.005$.

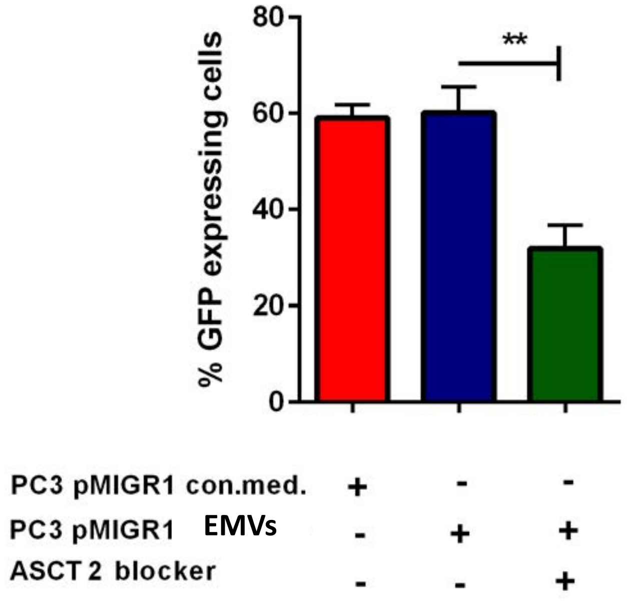

Figure S4. Inhibition of the GFP gene transfer to HUVEC cells with ASCT2 blocker. GFP transfer analysis for HUVEC cells incubated with the conditioned medium from GFP-retro PC3 cells, EMVs generated by these cells, and these EMVs applied in the presence of ASCT2 blocker (35 µl/ml). All results are shown as means ± SEM ($n \geq 3$). Level of significance relative to the data for EMVs from GFP-retro PC3 cells is shown as ** for $p<0.005$.

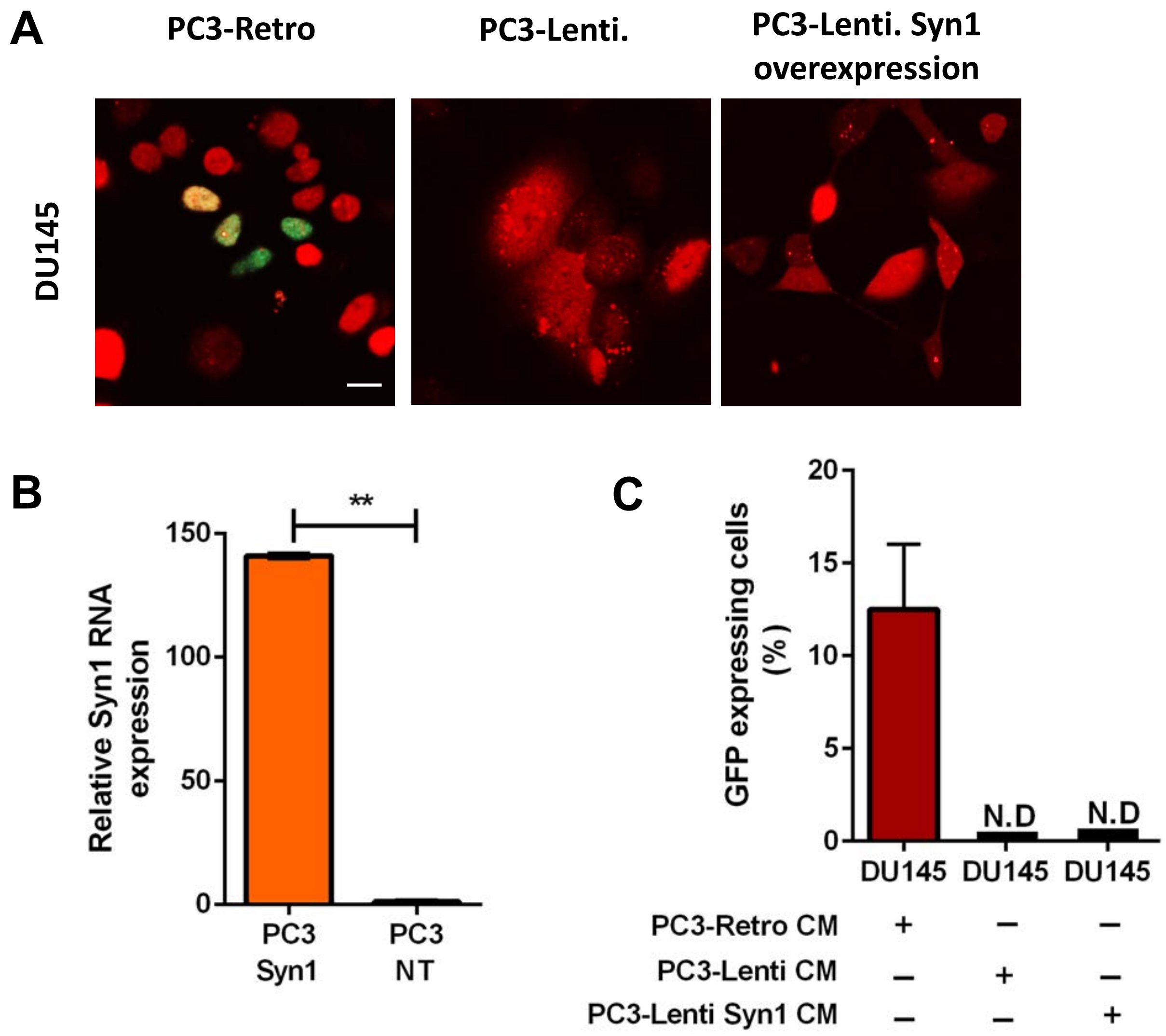

Figure S5. Syn1f expression in GFP-lenti PC3 cells did not induce GFP gene transfer to DU145 cells. Non-transduced DU145 cells were incubated in the conditioned media from GFP-retro PC3 cells, from GFP-lenti PC3 cells and from GFP-lenti PC3 cells expressing Syn1. (**A**) Representative images and (**B**) Overexpression of Syn1 at mRNA level in GFP-lenti PC3 cells cells was confirmed by qPCR (C) for the gene transfer. All results are shown as means ± SEM ($n \geq 3$). Levels of significance relative to the data for conditioned medium from GFP-retro PC3 cells expressing NT shRNA are shown as ** for p<0.005.

Figure 2B

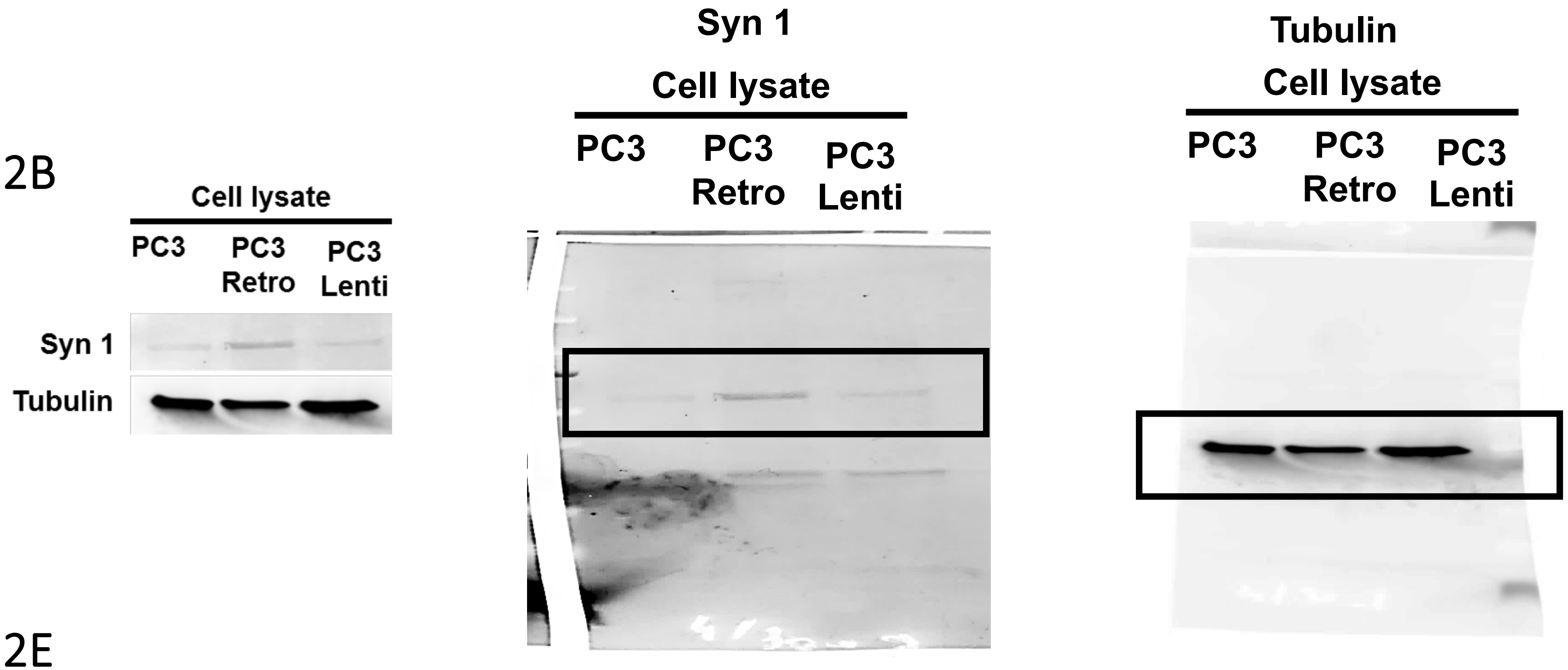

Figure 2E

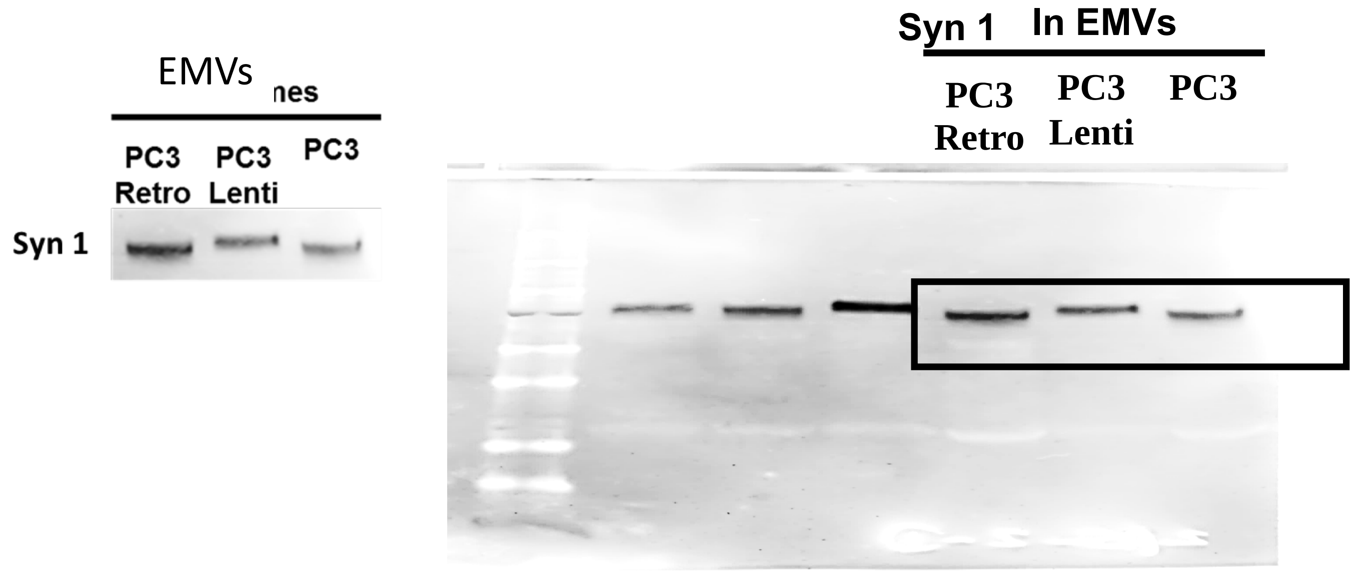

Figure S6. Full length gels for Fig. 2B and E

Figure 5B

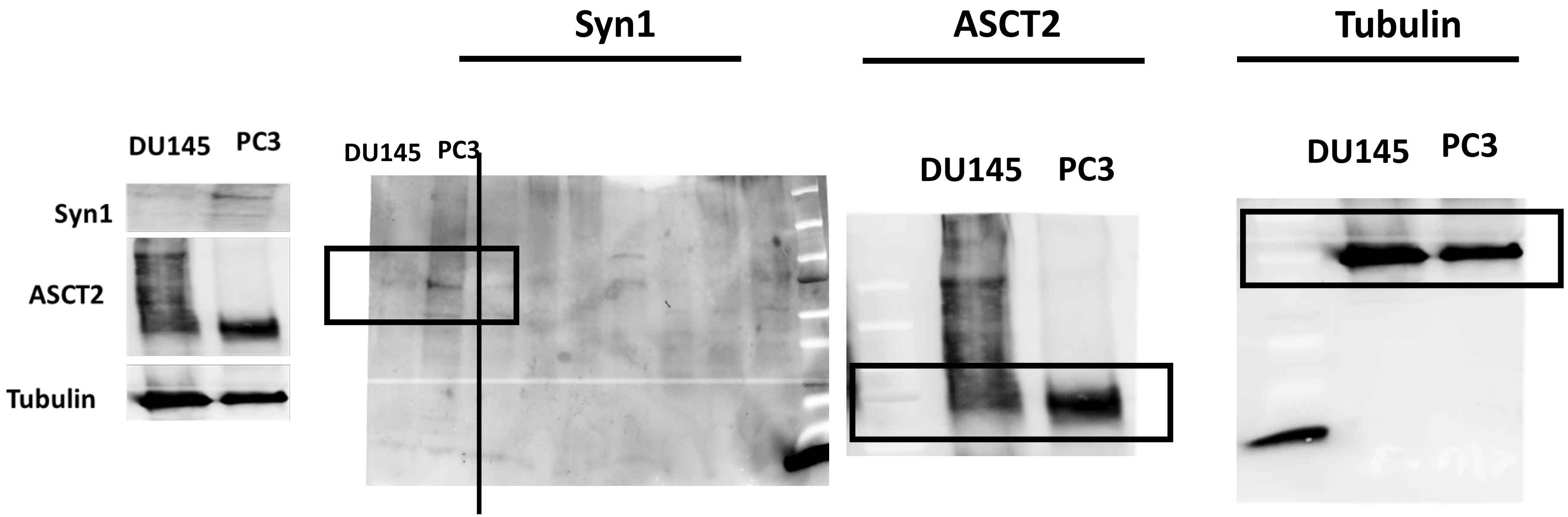

Figure S7. Full length gels for Fig. 5B

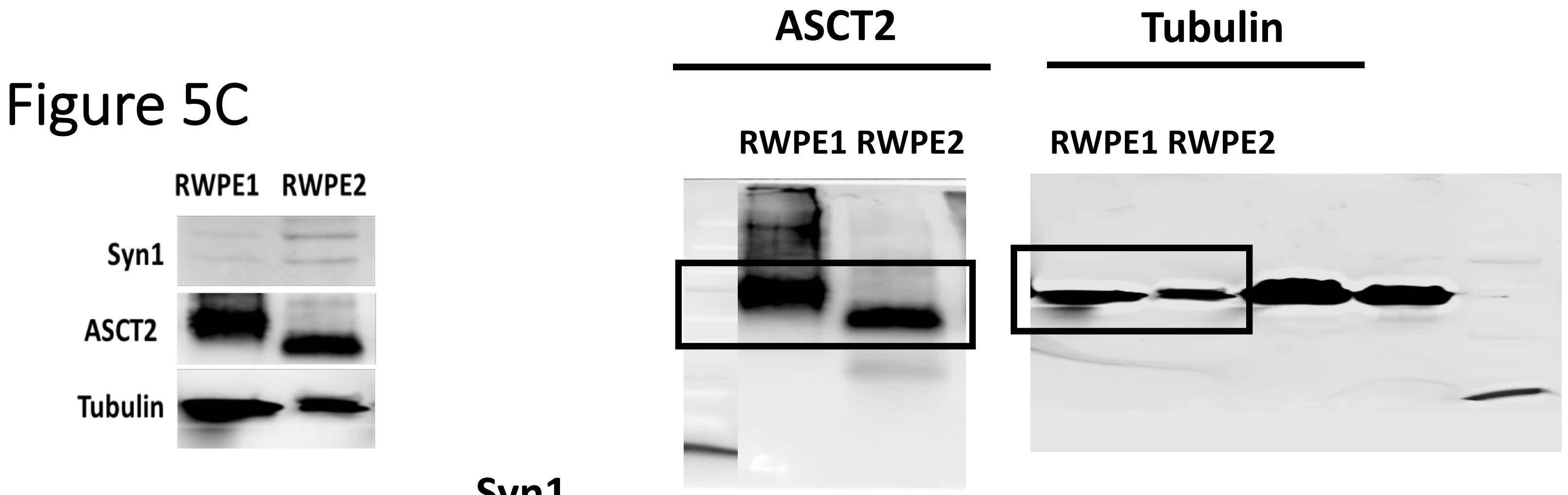

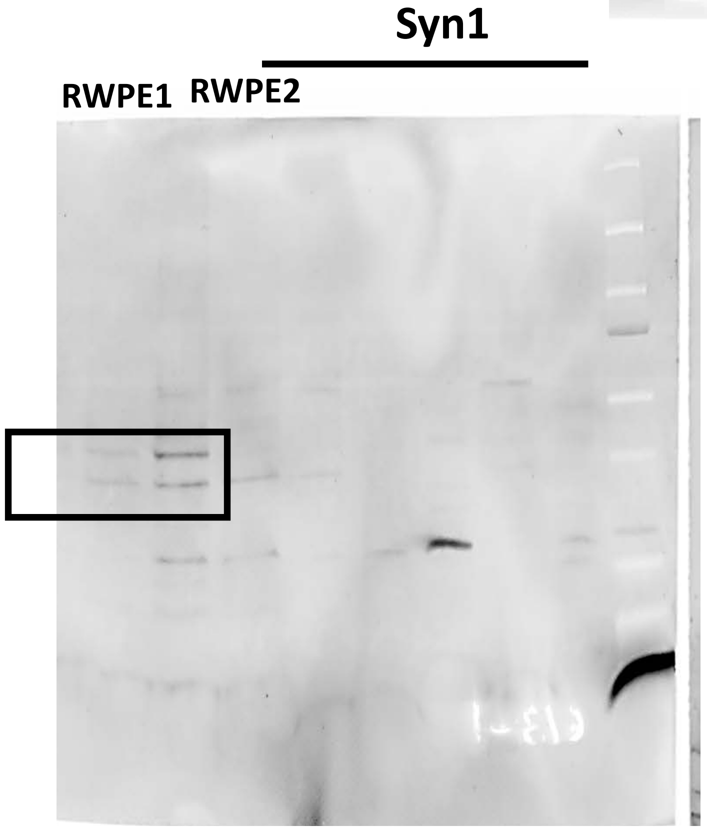

Figure S8. Full length gels for Fig. 5C

Figure 6A

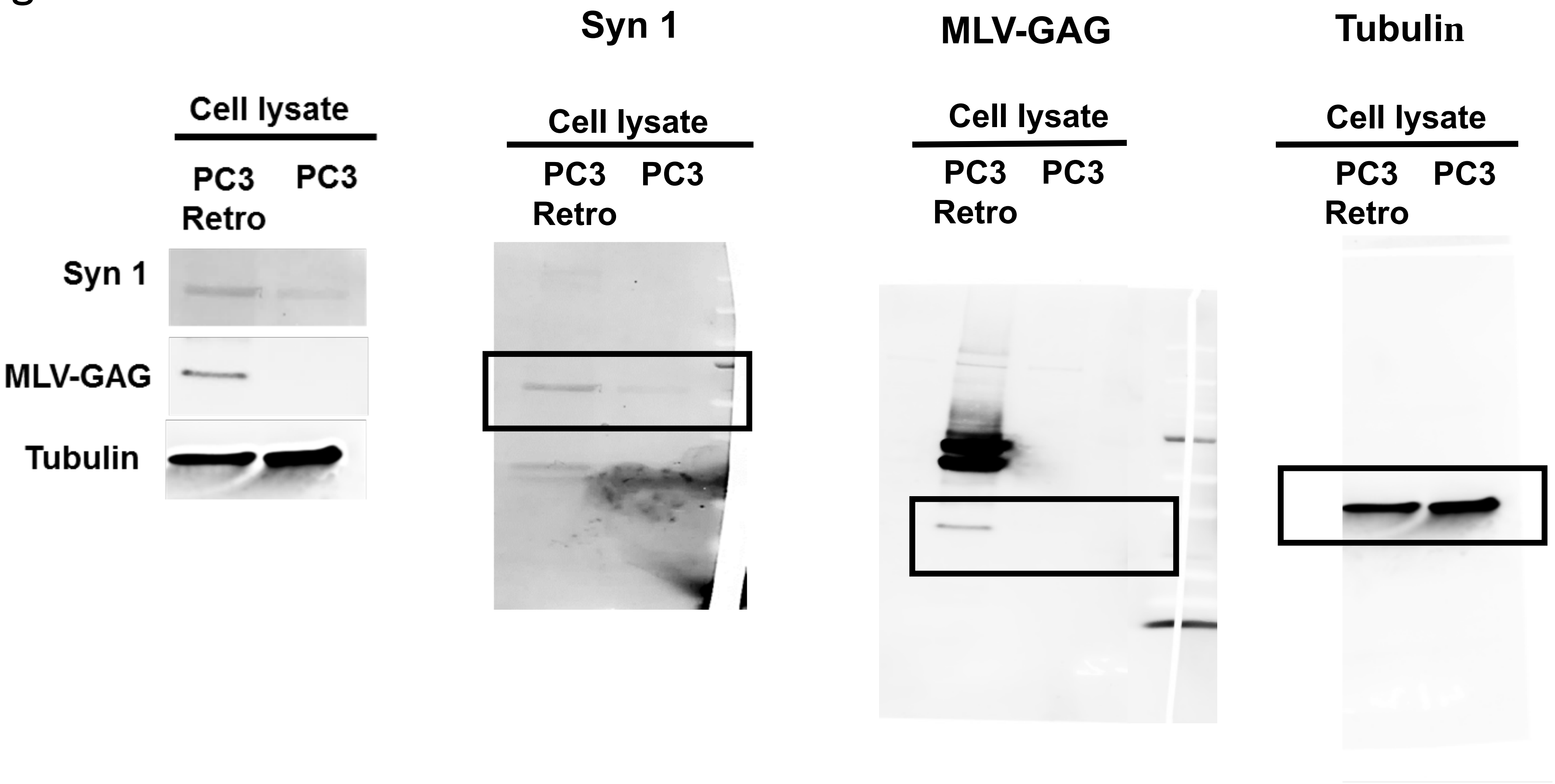

Figure S9. Full length gels for Fig. 6A

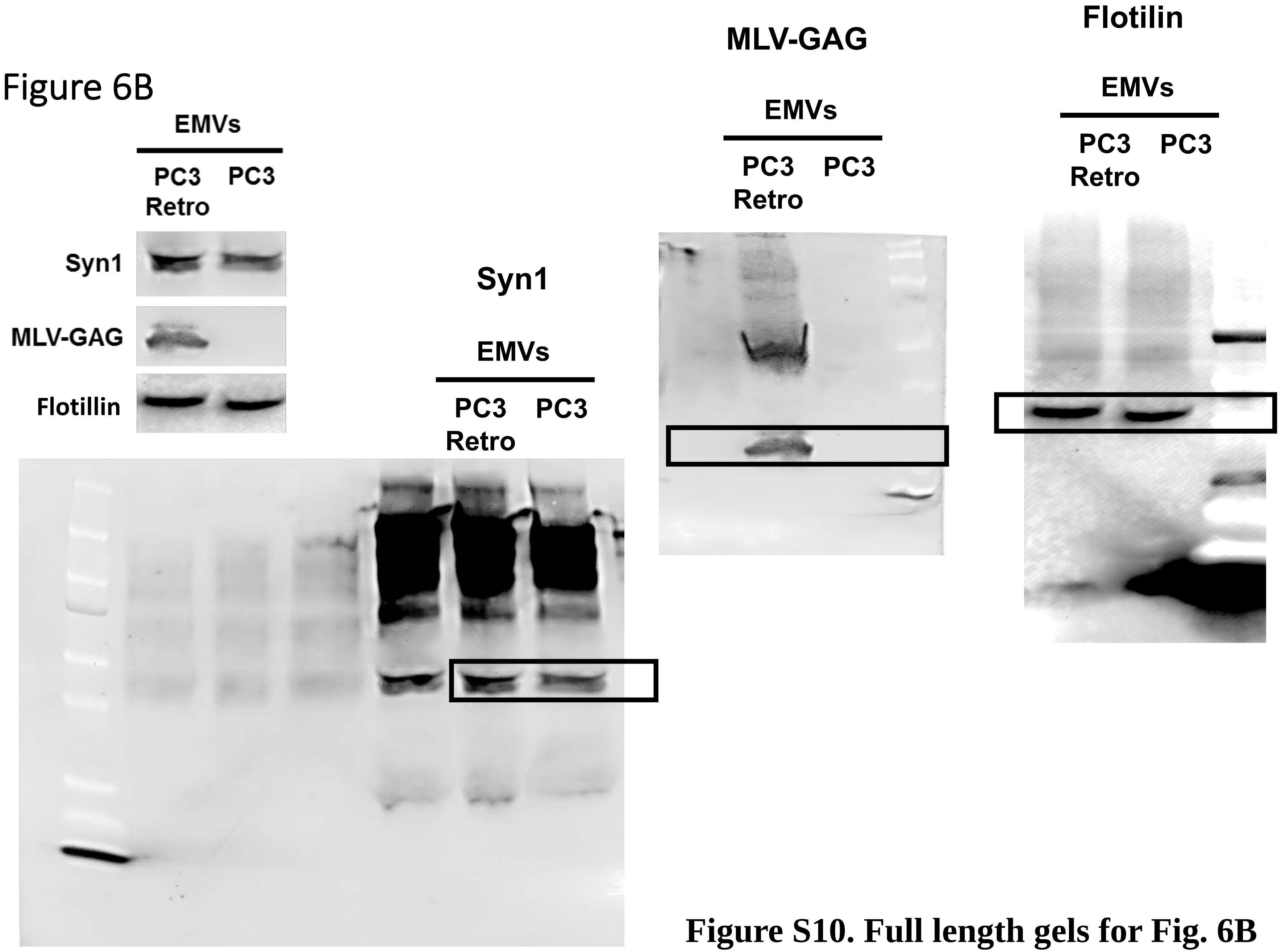

Figure S10. Full length gels for Fig. 6B

Fig. S1

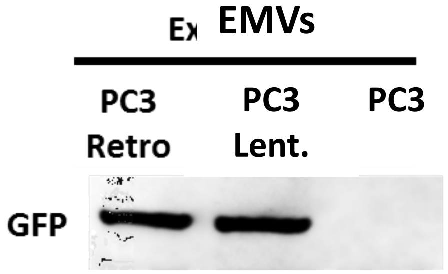

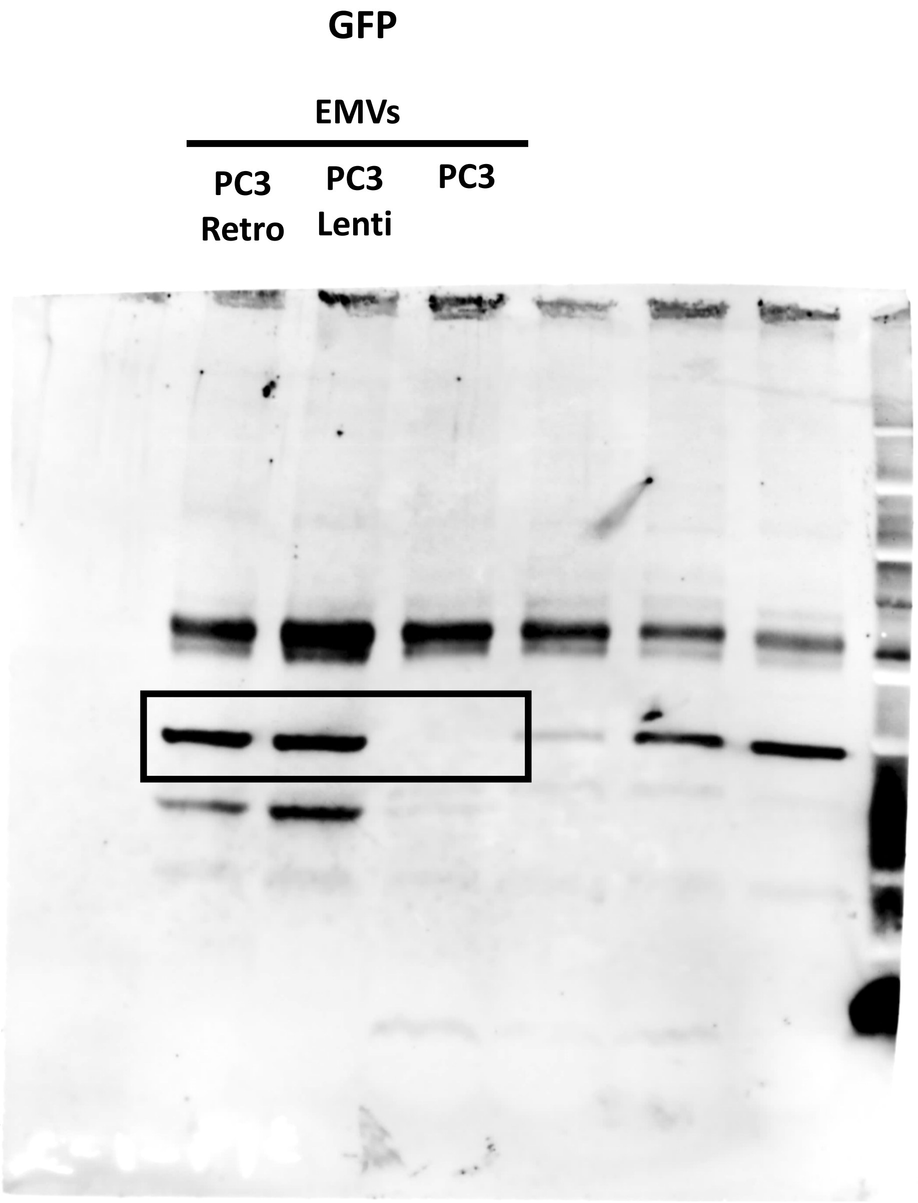

Figure S11. Full length gels for Fig. S1

Fig.S2A

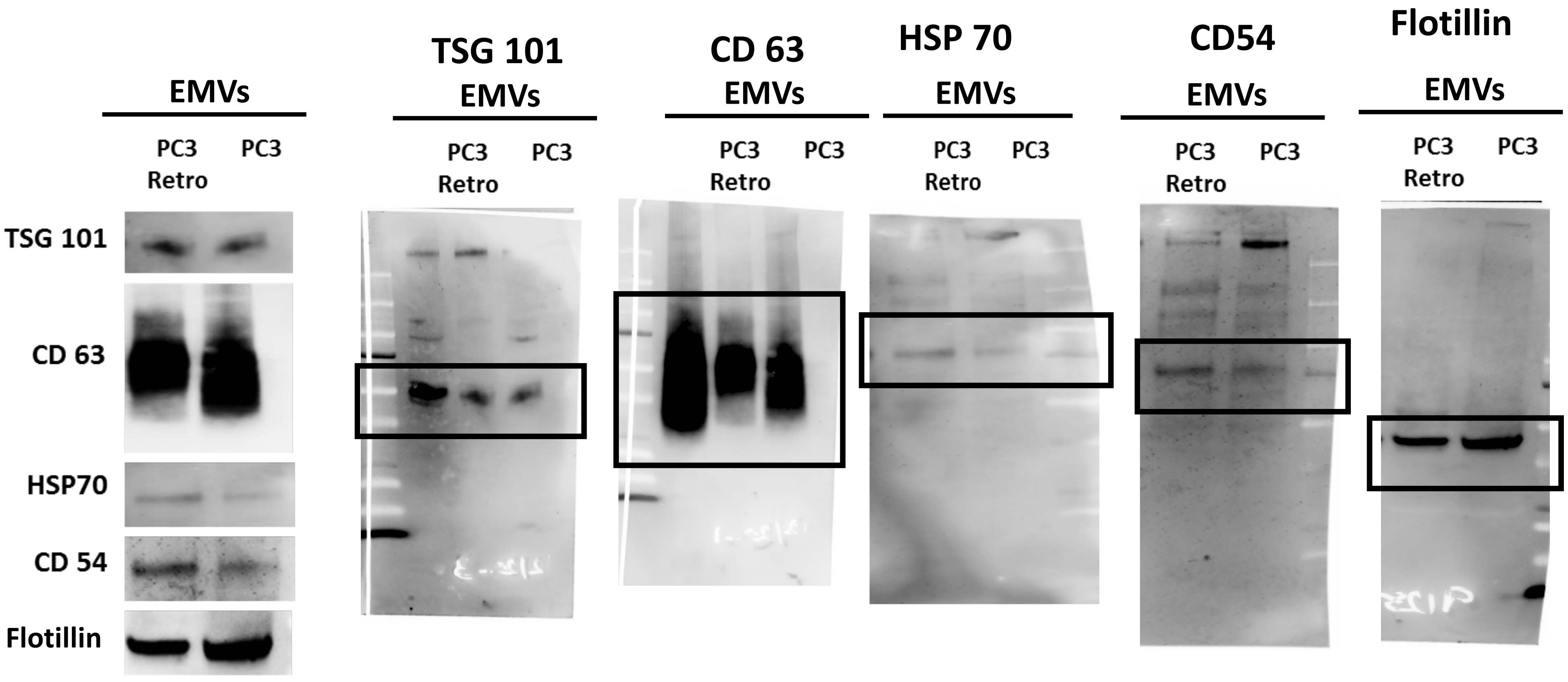

Figure S12. Full length gels for Fig. S2A

Fig.S3A

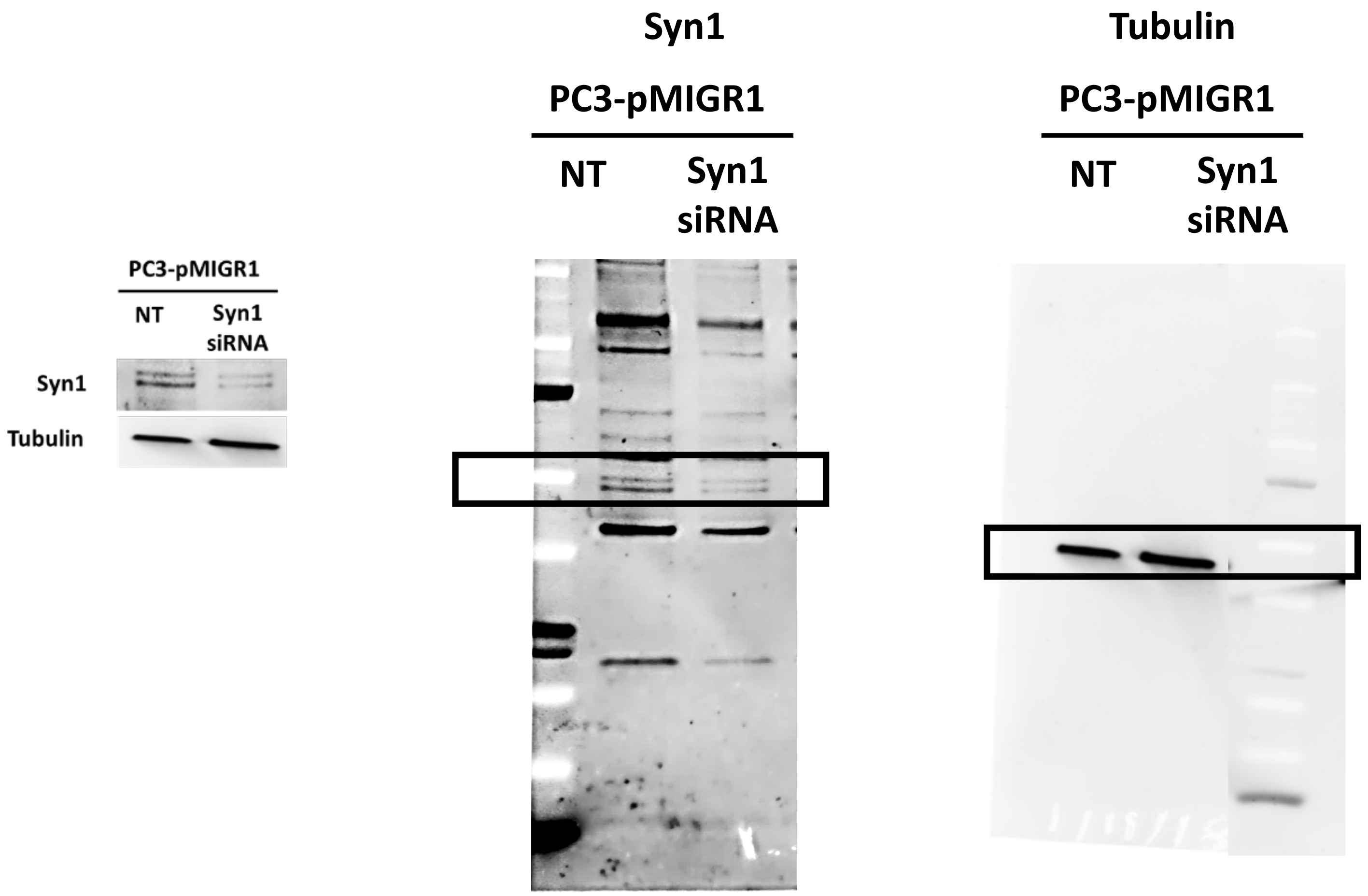

Figure S13. Full length gels for Fig. S3A
